# Prevalence of Malaria and Associated Risk Factors among the Community of Mizan-Aman Town and Its Catchment Area in Southwest Ethiopia

**DOI:** 10.1155/2022/3503317

**Published:** 2022-04-12

**Authors:** Tadesse Duguma, Abdulrezak Nuri, Yayeh Melaku

**Affiliations:** Department of Medical Laboratory Science, College of Health Science and Medicine, Mizan-Tepi University, Mizan-Aman, Ethiopia

## Abstract

**Background:**

Ethiopia is a Sub-Saharan African country with a high annual malaria case count, owing to the majority of the country's geography favoring vector rearing. As part of the country's prevention-based health policy, substantial efforts have been made to control and prevent malaria transmission. The objective of this study was to determine the prevalence of malaria and the associated factors in the community of Mizan-Aman and its catchment.

**Materials and Methods:**

From September to October 2021, a community-based cross-sectional survey was undertaken among the communities of Mizan-Aman town and its catchment area in Southwest Ethiopia. A pretested structured questionnaire was used to collect sociodemographic data, as well as a capillary blood sample from each study participant. Epi-data manager (v4.0.2.101) was used to enter the data and analyzed by SPSS version 25.0. A statistical significance was set at a *P* value of <0.05.

**Result:**

The study comprised a total of 412 people, of which 87 (21.1%) tested positive for malaria parasites, with a greater prevalence reported among those aged 25 to 34 years (5.8%). Individuals who lived near stagnant water were more likely to become infected with the malaria parasite (AOR = 8.996, 95% CI: 5.087-15.908) compared to those who lived further away, in warm climates, and those who did not use insecticide-treated bed nets were more susceptible to malaria parasite infection (AOR = 4.647, 95% CI: 1.257-17.184) compared to those who did use ITN and With (AOR = 0.466, 95% CI: 0.218-0.996 and AOR = 0.352, 95% CI: 0.206-0.604); participants with a history of antimalarial medication appear to have a protective function against malaria infection, respectively.

**Conclusion:**

The overall malaria prevalence in this study was 87 (21.1%), demonstrating that malaria remains a significant concern to the populations in the study area, with *Plasmodium falciparum* accounting for the vast majority of cases.

## 1. Background

Malaria is a serious public health issue that continues to cause illness and death. According to data from 85 malaria endemic countries, the worldwide malaria burden increased from 227 million cases in 2019 to 241 million cases in 2020, with the majority of the rise coming from countries in the African Region [1].

Due to service disruptions during the coronavirus pandemic, these regions were responsible for about 95% of malaria cases, with 14 million more cases and 47 000 more deaths reported worldwide than the previous year [2]. Malaria is one of the commonest diseases afflicting the impoverished in developing nations. Sub-Saharan Africa bears the brunt of the global malaria load, with the number of illnesses and deaths in the world being at an all-time high. It hurts people's health as well as economic development in many developing nations, especially in Sub-Saharan Africa [1, 3, 4].

As one of these countries, Ethiopia is plagued by malaria, which poses a serious threat to the country's health and economy. The disease's distribution pattern varies by climate, rainfall patterns, and altitude across the country [5].

*Plasmodium vivax*, *Plasmodium falciparum*, *Plasmodium malariae*, and *Plasmodium ovale* are the four most frequent species that cause human malaria, as well as a fifth parasitic species, *Plasmodium Knowles*, a monkey parasite [6]. *Plasmodium vivax* is responsible for the great majority of malaria infections, owing to its widespread distribution [7]. The only one of the four species that can be found in tropical, subtropical, and temperate climates is *P. vivax*. *Plasmodium falciparum* continues to infect people in tropical and subtropical areas, and it is the most common cause of a fatal type of malaria [8].

Malaria is spread by the *Anopheles* mosquito, which is the principal vector. *Anopheles arabiensis* is the primary vector, with secondary vectors such as *Anopheles phronesis*, *Anopheles funestus*, and *Anopheles nili* [9].

Human infection with *Plasmodium* species begins with a bite from a malaria-infected female *Anopheles* mosquito as the insect collects its blood meal. The number and variety of mosquitoes present in a specific region, as well as the temperature, determine the risk of infection [10]. Three climate zones in Ethiopia are conducive to malaria endemicity. Seasonal malaria transmission occurs in the “Kola” or hot zone below 1500 meters altitude, depending on local conditions, with moderate to high endemicity (46% of the territory). Malaria transmission is characterized by periodic outbreaks of unstable malaria occurring from unexpected climatic changes such as heavy rain or clouds in “Woina Dega” or temperate zone (46% of the land) between 1500 m and 2500 m altitude. The climatic area over 2500 m height, known as the “degas” or chilly zone, accounts for 8% of the country and is malaria-free [11]. Malaria infects about 75% of the country's territory, putting 68% of the population in danger of contracting the disease, which is estimated to kill 70,000 people each year [12, 13]. Malaria transmission in Ethiopia varies from season to season due to differences in altitude and the disease's longer duration of transmission in lowland areas, river basins, and valleys [14]. There are two malaria transmission seasons when the vectors are most abundant: September to December (major transmission) and April to May (minor transmission), both of which coincide with the major harvesting seasons [14].

Malaria epidemics are widespread in highland or highland fringe areas in Ethiopia, primarily 1,000 to 2,000 meters above sea level [15]. Changes in one or more climate variables, such as temperature, precipitation, wind, and sunshine, are examples of climate changes [13, 16]. Malaria cases number 2.9 million per year, with 4,782,000 deaths, and the rate of morbidity and mortality increases considerably during outbreaks [12].

Malaria remains a major public health problem despite significant successes and advances in improving people's health and lowering the disease's burden. It is one of the top ten major causes of sickness and mortality in a wide range of people, including children under the age of five and adults [17]. Malaria is also the leading cause of hospitalization, outpatient visits, and death [17]. To prevent further transmission, it is critical to screen and diagnose patients as soon as possible and treat them right away [16].

Ethiopia is still among the countries known for having very high malaria burden and in particular the study area (Mizan-Aman town community and its surrounding) was suffering a lot from this disease which is affecting health of the people living in that area as evidenced by the deaths of people in this area including children and pregnant women which consequently results in reduced working capacity and other day-to-day activities of the community as general. So, this study could help to provide additional insight for the country's government to strengthen prevention and control strategies to reduce and cease the health impact of the disease.

## 2. Materials and Methods

### 2.1. Study Setting and Period

The study was conducted at Mizan-Aman town and its catchment area from September to October 2021, which is found in the Benchi-Sheko zone, SNNPR region, Southwest Ethiopia.

### 2.2. Study Design and Population

A community-based cross-sectional study was conducted among Mizan-Aman town communities and its catchment area in Southwest Ethiopia. The study's source population was the Mizan-Aman town and its catchment area. The study populations were people who lived in selected households that were available during the data collection period and were chosen by a lottery system from each house in the research area. The study included participants with and without fever who had not used antimalarial medications or antibiotics in the previous month and were willing to participate and voluntarily supply blood samples.

### 2.3. Sample Size and Sampling Technique

The sample size was calculated using a single population proportion formula, and the following assumptions were considered 95% confidence interval; the estimated prevalence rate/proportion of malaria was taken as 50% since no similar study in the region. Based on those considerations, the sample size is calculated using the following formula:

(1) $$\begin{array}{c} \text{n}=\frac{{\left(Z_{\alpha /2}\right)}^{2}p\left(1-P\right)}{d^{2}}=\left(1.96\right)2\;0.5\left(1-0.5\right)/0.052=384 \end{array}$$

where *n* = minimum sample size, *P* = estimation prevalence rate of malaria 50%, *d* = margin of error (5%), and (Z*α*/2))^2^ = the standard normal variable.

After adding 10% for possible nonresponse, the final sample size became 384 + 384(0.1) = 422.

Study participants who fulfilled the eligibility criteria were recruited by a systematic random sampling technique considering the population density and proximity of the households to each other. All individuals with and without signs and symptoms of malaria and available during the study period were included. One study subject from each of the selected households was selected randomly to participate in the study. If the selected study participants were an infant/child, guardian/caregiver, or parents, they were interviewed instead. Individuals who were on antimalarial drug treatment, antibiotics therapy within the past 30 days before recruitment, and unable to provide blood samples were excluded.

### 2.4. Data Collection Tools and Procedure

The data were collected using structured questionnaires modified from previous research [18] that included both sociodemographic and risk factor characteristics. The finger was cleansed with 70% alcohol-soaked cotton before the blood sample was taken. Each study participant had a drop of blood, approximately 50 *μ*L (capillary blood from the fingertip), taken by finger prick, and both thick and thin films were prepared according to the standard operating procedures.

### 2.5. Data Collection Process and Management

Medical laboratory workers collected data after receiving a two-day instruction on the study's goals and how to gain informed consent from the study participants. For data collection, three medical laboratory technologists were recruited, as well as two supervisors to help with the operations.

### 2.6. Malaria Microscopy

Giemsa was used as it is the standard stain used for staining of blood films for malaria diagnosis, and blood films microscopy was done since it remains a basic technique and the gold standard for the diagnosis of malaria in resource limited settings. Details of the test procedure and principle are shown at the end of the manuscript (Appendix).

### 2.7. Data Quality Assurance

The data collectors described the data gathering techniques, technologies used, and how to tackle ethical considerations. Before the start of the main study, a pretest was conducted with 5% of the sample size at Tepi town, which is 50 kilometers away from the study area. Data collectors received two days of training on how to collect data. During data collection, the questionnaire was translated into the respondent's native language. The supervisors and the investigators kept a close eye on the data collection process to ensure that everything was done correctly.

### 2.8. Data Processing and Analysis

To detect malaria parasites, the blood films were stained with a 10% Giemsa working solution and examined microscopically using a 100X oil immersion objective, and a thin blood film was prepared according to the SOPs. Epi-data manager (v4.0.2.101) was used to enter the data, and SPSS version 25.0 was used to analyze it. To see if there was a link between the outcome variable and the risk factors, researchers used descriptive statistics and bivariate and multivariable logistic regression.

## 3. Results

### 3.1. Sociodemographic Characteristics of the Study Subjects

A large number of 412 people who took part in the study were infected with malaria parasites. Malaria was recorded in people of all ages; however, the infection rate was highest in people aged 25 to 34. Malaria parasite prevalence was high among low-income persons, possibly due to people's inability to afford mosquito bed nets. The survey included people from various occupations, with housewives and students contributing disproportionately greater numbers of malaria cases. (Table 1).

### 3.2. Prevalence of Malaria

**Figure 1:**
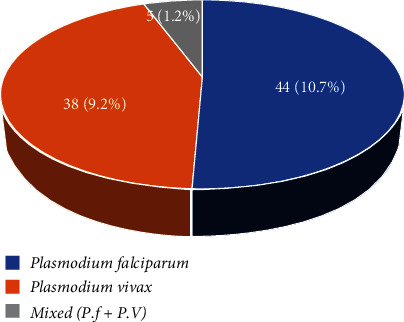
Types of plasmodium species and their distribution.

Out of the 412 participants who took part in the study, 87 (21.1%) were infected with malaria parasites, with *Plasmodium falciparum* and *Plasmodium vivax* being the two species that were found (Figure 1).

### 3.3. Possible Risk Factors for Malaria Infection

#### 3.3.1. ITNs Availability, ITNs Utilization, Insecticide Spray, Climate Condition, and Stagnant Water Presence

Data was collected from 412 study participants to look into the role of various risk factors in malaria prevalence. As a result, more than three-quarters of them lacked access to insecticide-treated bed nets (ITNs), which contributes to the bulk of malaria cases in our study. Furthermore, nearly two-thirds of those polled said that their home was not sprayed with insecticide chemicals. Individuals who live near stagnant water and in hotter climates were found to be more susceptible to malaria infection (Table 2).

## 4. Discussion

Despite significant financial investments and efforts made both locally and globally to reduce malaria transmission, the disease continues to be the most serious threat to people's health in Sub-Saharan Africa, particularly Ethiopia. This study had recruited a total of four-hundred twenty-two participants; however, only 412 of them with complete data (interviews and blood samples) were included in the analysis, resulting in a none-response rate of (2.4%). The study included two hundred one male and two hundred eleven female participants, and having a low income seems to contribute the most to malaria prevalence in this study (18.7%). Moreover, a quarter of the study, participants were students, who were the second most category after housewives in terms of contribution to malaria positivity rate compared to other occupations in this study.

Overall, eighty-seven (21.1%) of the study participants had malaria parasites, with *Plasmodium falciparum* (10.7%) having the highest prevalence, followed by *Plasmodium vivax* (9.2%). A study from Butajira, Ethiopia's south-central region, found a *Plasmodium falciparum* prevalence of 12.4%, which was similar to the current study [19]. The prevalence of malaria was found to be greater in age groups between 25 and 34 (5.8%) than in other age groups in the current study, which is consistent with a recent study conducted in Ghana that indicated the prevalence of malaria is more common among people of similar age [20]. Malaria was more common among illiterates (12.9%) than among literates (8.3%). This could be attributed to a difference in the amount of information about preventive strategies between literates and those with no education. Three hundred thirty (80.1%) of the study participants said that their home was not treated with insecticides/chemicals, which could explain the comparatively high malaria prevalence (16.3%) compared to those whose homes were sprayed (4.9%) from the total positive cases. Malaria prevalence was found to be greater (11.4%) compared to urban dwellers (9.7%**).** This could be related to the projected decreased exposure and accessibility to the medium of communication in rural communities compared to metropolitan dwellers (9.7%). Malaria cases were found to be more prevalent in warmer/“Kolla” climate conditions (74%), followed by ten (2.4%) and three (0.7%) for medium/Woina Dega and colder/Dega climate conditions, respectively. Although the study participants agreed that “the use of ITNs is a powerful vector control tool for preventing malaria transmission and thus reducing the prevalence of the disease elsewhere in the country where malaria is endemic,” only 20.4 percent of them had ITNs in their homes, and ITN ownership by itself is not a guarantee for its use, as evidenced by the finding that more than three-quarters of the study participants (82.5%) did not utilize a bed net (Table 2).

In this study, it was discovered that the use of ITNs differs significantly from a study from Kenya, which found that approximately (92.11%) of households use mosquito bed nets, and malaria prevalence is lower among households that use mosquito bed nets (8.05%) than those who do not use mosquito nets (23.11%) [21].

The use of ITNs is considered one of the parasite's protective mechanisms; nonetheless, the frequency of malaria among individuals who did not use ITNs (17.5%) was significantly greater than among those who did. More than three-quarters of the study participants said that they did not have an ITN in their home, which is evidenced by the high malaria infection observed among those ITN nonusers. Sixty-two (15.0%) participants whose blood film examination revealed the presence of malaria parasites responded that there was stagnant water nearby their home, as indicated by the high rates of malaria infection among nonusers of ITNs. Sixty-two (15.0%) of those whose blood film tests confirmed the presence of malaria parasites said that there was stagnant water near their residence. People who lived near stagnant water were nearly nine times more likely to contract malaria (AOR = 8.996, 95% CI: 5.087-15.908), while those who lived in hot climates were four times more likely to contract *Plasmodium* infection (AOR = 4.647, 95% CI: 1.257-17.184). People who now have access to ITNs have also been found to be more susceptible to malaria parasite infection (AOR = 4.161, 95% CI: 1.760-9.836). Using an ITN and having a history of antimalarial medication, on the other hand, appears to protect against malaria infection (AOR = 0.466, 95% CI: 0.218-0.996) and (AOR = 0.352, 95% CI: 0.206-0.604), respectively.

The malaria prevalence found in this study (21.1%, 87/412) was lower than other studies conducted in different parts of the world, such as various areas of Nigeria((82.72%, 426/515), (64.9%, 227/350), (51%, 51/100), (41.6%, 106/255), (35.7%, 419/1173)), India (22-26, 36.6%), Malaysia (33.6%, 410/1222) [27, 28], Kenya (325/1158, 28%), Rwanda (22.8%, 175/769) [29], East Wollega Ethiopia (21.2%, 26679/125917)(49.4%, 156/316) [18, 31], North-Western Ethiopia (32.6%, 33,43/102520),(29.0%, 61/210)), South Ethiopia (28.1%, 91/324^)^ [32, 33], [17], East Shewa Ethiopia (20.5%, 170/830)(25%, 204/810) [34, 35], and Arba Minch South Ethiopia (22.1%, 60/271) [36] and significantly higher than studies from West Ethiopia (10.2%, 51/498) [37], South Ethiopia (6.1%, 28/461) [39, 40], and North-West Ethiopia(7.3%, 296/4077), (3.5%, 26/735). These variances could be related to differences in geographical location and climate conditions of the study participants. The result of this study showed that malaria is still a serious public health concern in different parts of the country so the information obtained from this study can be used to device means (control and prevention strategies) to stop further suffering of the community from this disease.

## 5. Limitations

The finding of this study could be better given that the testing was done using advanced molecular techniques like polymerase chain reaction (PCR) and loop-mediated isothermal amplification (LAMP) which have higher detection capacity compared to light microscopy.

## 6. Conclusions

The overall malaria prevalence in the research was 87 (21.1%), indicating that malaria remains a serious public health concern in the area, with *Plasmodium falciparum* being the most common species. Malaria was more common in people between the ages of 25 and 34. (5.8%). Providing health education that enhances people's knowledge of the disease and changing community ITN usage behaviors may assist to stem the spread of the disease.

## Figures and Tables

**Table 1:** Distribution of malaria by sex, age, educational status, income, and occupational status among the communities of Mizan-Aman town and its catchment area, 2021 (*n* = 412).

Sociodemographic variables		Malaria		
		Positive *N* (%)	Negative *N* (%)	Total *N* (%)
Sex	Male	44 (10.7)	157 (38.1)	201 (48.8)
	Female	43 (10.4)	168 (40.8)	211 (51.2)
Age in year/s	0-4	7 (1.7)	46 (11.2)	53 (12.9)
	5-14	13 (3.2)	60 (14.6)	73 (17.7)
	15-24	14 (3.4)	70 (17.0)	84 (20.4)
	25-34	24(5.8)	76 (18.4)	100 (24.3)
	35-44	18 (4.4)	45 (10.9)	63 (15.3)
	45-55	10 (2.4)	25 (6.1)	35 (8.5)
	>55	1 (0.2)	3 (0.7)	4 (1.0)
Educational status	Illiterate	53 (12.9)	166 (40.3)	219 (53.2)
	Literate	34 (8.2)	159 (38.6)	193 (46.8)
Income	Low	62 (18.7)	100 (30.1)	162 (48.8)
	Middle	17 (5.1)	107 (32.2)	124 (37.3)
	High	7 (2.1)	39 (11.7)	46 (13.9)
Occupation status	Farmer	13 (3.2)	68 (16.5)	81 (19.7)
	Merchant	1 (0.2)	12 (2.9)	13 (3.2)
	Government employee	8 (1.9)	15 (3.6)	23 (5.6)
	Student	23 (5.6)	104 (25.2)	127 (30.8)
	Housewife	26 (6.3)	73 (17.7)	99 (24.0)
	Daily worker	15 (3.6)	40 (9.7)	55 (13.3)
	Other	1 (0.2)	13 (3.2)	14 (3.4)
Total		87 (21.1)	325 (78.9)	412 (100.0)

**Table 2:** Bivariate and multivariable analysis result for possible factors associated with malaria infection among the communities of Mizan-Aman town and its catchment area, 2021 (*n* = 412).

Variables		Malaria			OR (95% CI)	
		Positive *N* (%)	Negative *N* (%)	Total *N* (%)		
					COR	AOR
Educational status	Illiterate	53 (12.9)	166 (40.3)	219 (53.2)	1.493 (0.922, 2.419)	1.011 (0.571, 1.788)
	Literate	34 (8.2)	159 (38.6)	193 (46.8)	Ref	Ref
ITN availability	Yes	9 (2.2)	75 (18.2)	84 (20.4)	Ref	Ref
	No	78 (18.9)	250 (60.7)	328 (79.6)	0.385 (0.184, 0.803)^∗∗^	4.161 (1.760, 9.836)^∗∗^
ITN utilization	Yes	15 (3.6)	57 (13.8)	72 (17.5)	Ref	Ref
	No	72 (17.5)	268 (65.0)	340 (82.5)	0.980 (0.524, 1.831)	0.466 (0.218, 0.996)^∗∗^
Presence of stagnant water	Yes	62 (15.0)	69 (16.7)	131 (31.8)	0.109 (0.064, 0.186)	8.996 (5.087, 15.908)^∗∗^
	No	25 (6.1)	256 (62.1)	281 (68.2)	Ref	Ref
House sprayed with insecticide	Yes	30 (7.3)	110 (26.7)	140 (34.0)	Ref	Ref
	No	57 (13.8)	215 (52.2)	272 (66.0)	1.266 (0.715, 2.241)	0.643 (0.340, 1.218)
History of anti-malarial treatment	Yes	32 (7.8)	60 (14.6)	92 (22.3)	Ref	Ref
	No	55 (13.3)	265 (64.3)	320 (77.7)	2.570 (1.531, 4.314)^∗∗^	0.352 (0.206, 0.604)^∗∗^
Pregnancy	Pregnant	5 (1.2)	14 (3.4)	19 (4.6)	Ref	Ref
	Non-pregnant	38 (9.2)	154 (37.4)	192 (46.6)	1.136 (0.697, 1.850)	0.847 (0.416, 1.727)
Residence	Urban	40 (9.7)	165 (40.0)	205 (49.8)	Ref	Ref
	Rural	47 (11.4)	160 (38.8)	207 (50.2)	0.825 (0.514,.326)	1.153 (0.678, 1.960)
The climate condition of the study setting	Warm (Kolla)	77 (18.7)	261 (63.3)	338 (82.0)	0.269 (0.079, 0.920)^∗∗^	4.647 (1.257, 17.184)^∗∗^
	Medium (Woina Dega)	9 (2.2)	54 (13.1)	63 (15.3)	1.608 (0.413, 6.259)	0.850 (0.202,3.576)
	Colder (Dega)	1 (0.2)	10 (2.4)	11 (2.7)	Ref	Ref
Total		87 (21.1)	325 (78.9)	412 (100.0)		

Abbreviations: AOR, adjusted odds ratio; COR, crude odds ratio; OR, odds ratio, (∗∗) indicates significance at *p* < 0.05. Ref represents the reference category during analysis.

## Data Availability

The data related to this research can be obtained from the corresponding author upon reasonable request.

## Appendix

### A. Malaria Microscopy

#### A.1. Collection of Finger Prick Blood and Preparation of Thick and Thin Blood Films

##### A.1.1. Procedure

Prepare a glass slide that has been precleaned and the other materials needed for blood collection. Select the 3^rd^ or the 4^th^ finger from the thumb (or big toe for infants). Hold the patient's hand, palm facing upwards, and clean the selected finger with 70% ethanol or alcohol swab. Use firm strokes to remove dirt and oils from the ball of the finger and to stimulate blood circulation. Let the alcohol dry from the finger.

- Prick the finger (or big toe) with a new, sterile lancet for every patient. Apply gentle pressure to the finger (or toe), and express the first drop of blood. Wipe the first drop of blood with dry cotton, making sure that no cotton strands remain on the finger that might stick to the blood
- Make both thick and thin blood films (for one patient) on the frosted side of the same slide
- To prepare the thin film, place the edge of a clean “spreader” slide at a 45° angle in front of the blood drop intended for the thin film
- Slowly pull the “spreader” back until it touches the drop of blood and it spreads along the edge of the “spreader”
- Rapidly push the “spreader” forward (away from the center) in a smooth, continuous motion, until the spreader leaves the bloody part of the slide leaving a “feathery” end for the thin film
- With the corner of the same “spreader” used for making the thin film, make the thick film by swirling the 3 drops of blood together forming a circle of about 1.2 cm diameter

### B. Staining of Malaria Blood Films

In order to prepare staining of malaria blood films with Giemsa, there are three steps to follow: First, prepare the buffered water to pH of 7.2 for use in the preparation of Giemsa stain solution. Second, prepare the working solution of Giemsa stain for routine staining of malaria blood film. Then, malaria blood films are staining with Giemsa stain.

### C. Principle

A freshly prepared working solution of Giemsa, made from well-prepared stock and diluted with water buffered to pH 7.2, is recommended to achieve optimal staining quality of malaria blood films. Giemsa stock solution prepared for the national programmed is standardized to minimize the need for frequent adjustment of SOPs for staining.

A properly stained blood film is critical for malaria diagnosis especially for precise identification of malaria species. The use of Giemsa stain is the recommended and most reliable procedure for staining thick and thin blood films. Giemsa solution is composed of eosin and methylene blue (azure). The eosin component stains the parasite nucleus red, while the methylene component stains the cytoplasm blue. The thin film is fixed with methanol. Dehemoglobinization of the thick film and staining takes place at the same time during the process.

An ideal pH of 7.2 is required to demonstrate stippling of the parasites to allow proper species identification.

### D. Examining thick and thin malaria blood films

In the thick film, the red blood cells (RBCs) are lysed and dehemoglobinized, while the malaria parasites are left intact and concentrated allowing their proper detection and identification. In the thin film, when fixed with absolute methanol, the RBCs retain their original morphology and, if the malaria parasites are present, become visible inside the cells. It is critical that malaria diagnosis is based on well-prepared thick and thin malaria blood films to ensure correct speciation and accurate estimation of parasite density.

Detection and identification of the *Plasmodium* species and stages:

- Place the Giemsa-stained blood film to be examined on the microscope stage
- Position the thick film in line with the objective lens
- Switch on the microscope and adjust the light source optimally by looking through the ocular and the ×10 objective (low power)
- Place a drop of immersion oil on the thick film, and allow it to spread
- To avoid cross contamination, ensure that the immersion oil applicator never touches the slide
- Scan the blood film for parasites and blood elements. Select part of the film that is well stained and has evenly distributed white blood cells
- Switch to ×100 oil immersion objective over the selected portion of the thick film
- Raise the mechanical stage until the objective lens gently touches the immersion oil but not the slide
- Examine the slide in a systematic manner. Start at the top left end of film, and begin at the periphery of them field, and then move horizontally to the right, field by field. Alternatively, from the top left end of the film, move vertically downwards to the next adjacent fields. When the other end of the film is reached, move the slide to the right, and then go upwards to the adjacent fields, and so forth

## Abbreviations

AOR: Adjusted odds ratio
COR: Crude odds ratio
ITNs: Insecticide-treated nets
LAMP: Loop-mediated isothermal amplification
OR: Odds ratio
PCR: Polymerase chain reaction
SPSS: Statistical package for social sciences
WHO: World Health Organization.

## References

[1] World Health Organization (2020). World malaria report 2020. 20 years of global progress and challenges.

[2] World Health Organization (2021). *World malaria report 2021*.

[3] Barofsky J., Claire C., Tobenna A., Farshad F. (2011). The economic effects of malaria eradication: evidence from intervention in Uganda. *Program on the Global Demography of Aging Working Paper*.

[4] Castillo R., McIntyre M., Barnes K. D. (2008). The household burden of malaria in South Africa and Mozambique: is there a catastrophic impact?. *Tropical Medicine & International Health*.

[5] Legesse Y., Ayalew T., Tefera B., Kora T. (2007). Knowledge, attitude, and practice about malaria transmission and its preventive measures among households in urban areas of Assosa Zone, Western Ethiopia. *Ethiopian Journal of Health Development*.

[6] Krief S., Pacheco A. E., Mugisha M. A. (2010). On the diversity of malaria parasites in African apes and the origin of plasmodium falciparum from bonobos. *PLoS Pathogens*.

[7] Kotepui M., Kotepui K., Milanez G., Masangkay F. (2020). Prevalence and risk factors related to poor outcome of patients with severe Plasmodium vivax infection: a systematic review, meta-analysis, and analysis of case reports. *BMC Infectious Diseases*.

[8] Deress T., Girma M. (2019). Plasmodium falciparum and Plasmodium vivax prevalence in Ethiopia: a systematic review and meta-analysis. *Malaria Research and Treatment*.

[9] Mariam M. G., Bekele W., Balkew M. (2017). *Federal democratic republic of Ethiopia ministry of health*.

[10] Burkett-Cadena N. D., Vittor A. Y. (2018). Deforestation and vector-borne disease: forest conversion favors important mosquito vectors of human pathogens. *Basic and Applied Ecology*.

[11] Alemu A., Tsegaye W., Golassa L., Abebe G. (2011). Urban malaria and associated risk factors in Jimma town, South-West Ethiopia. *Malaria Journal*.

[12] Girum T., Shumbej T., Shewangizaw M. (2019). Burden of malaria in Ethiopia, 2000-2016: findings from the Global Health Estimates 2016. *Tropical Diseases, Travel Medicine and Vaccines*.

[13] World Health Organization (2016). World malaria report 2015.

[14] Berhe B., Mardu F., Legese H., Negash H., Negash H. (2019). Seasonal distribution and seven year trend of malaria in North West Tigrai: 2012–2018, Ethiopia; 2019. *Tropical Diseases Travel Medicine and Vaccines*.

[15] Deressa W., Ali A., Berhane Y. (2006). Review of the interplay between population dynamics and malaria transmission in Ethiopia. *Ethiopian Journal of Health Development*.

[16] Ethiopian Public Health Institute (2015). *National Research Institute, and Federal Minister of Health. Ethiopia national malaria indicator survey*.

[17] Belete E., Roro A. (2016). Malaria prevalence and its associated risk factors among patients attending Chichu and Wonago Health Centres, South Ethiopia. *Journal of Research in Health Sciences*.

[18] Bidu K., Babure Z. (2019). Prevalence of malaria and associated factors among febrile patients visiting Kalala Health Center in Haro Limmu Woreda, East Wollega Zone, Western Ethiopia. *Epidemiology*.

[19] Woyessa A., Deressa W., Ali A., Lindtjørn B. (2012). Prevalence of malaria infection in Butajira area, south-central Ethiopia. *Malaria Journal*.

[20] Boadu I., Nsemani W., Ubachukwu P., Okafor F. (2020). Knowledge and prevalence of malaria among rural households in Ghana. *Journal of Community Medicine Health Education*.

[21] Sultana M., Sheikh N., Mahumud R., Jahir T., Islam Z., Sarker A. (2017). Prevalence and associated determinants of malaria parasites among Kenyan children. *Tropical Medicine and Health*.

[22] Oladele O., Onuoha S., Hamafyelto H. (2018). Prevalence of malaria infection among patients attending Murtala Muhammed specialist hospital Kano, Nigeria. *African Journal of Clinical and Experimental Microbiology*.

[23] Omoya F., Ajayi K. (2020). Prevalence of Malaria among Febrile Patients attending Government Hospitals in Ondo State, South-West Nigeria. *American Journal of Epidemiology & Public Health*.

[24] Dogara M., Ocheje J. (2016). Prevalence of malaria and risk factors among patients attending Dutse General Hospital, Jigawa State, Nigeria. *International Research Journal of Public and Environmental Health*.

[25] Fana S., Bunza M., Anka S., Imam A., Nataala S. (2015). Prevalence and risk factors associated with malaria infection among pregnant women in a semi-urban community of north-western Nigeria. *Infectious Diseases of Poverty*.

[26] Umaru M., Uyaiabasi G. (2015). Prevalence of malaria in patients attending the general hospital Makarfi, Makarfi Kaduna–State, North-Western Nigeria. *American Journal of Infectious Diseases and Microbiology*.

[27] Dayanand K., Punnath K., Chandrashekar V. (2017). Malaria prevalence in Mangaluru city area in the southwestern coastal region of India. *Malaria Journal*.

[28] Ramdzan A., Ismail A., Mohd Zanib Z. S., Ismail A., Zanib Z. M. (2020). Prevalence of malaria and its risk factors in Sabah, Malaysia. *International Journal of Infectious Diseases*.

[29] Jenkins R., Omollo R., Ongecha M. (2015). Prevalence of malaria parasites in adults and its determinants in a malaria-endemic area of Kisumu County, Kenya. *Malaria Journal*.

[30] Rulisa S., Kateera F., Bizimana J. (2013). Malaria prevalence, spatial clustering and risk factors in a low endemic area of Eastern Rwanda: a cross-sectional study. *PLoS One*.

[31] Tadele G., Samuel A., Adeba E. (2014). Replacement of Long Lasting Insecticide Treated Nets in Malarious Kebeles of Gida Ayana District, East Wollega Zone, Ethiopia. *Science, Technology and Arts Research Journal*.

[32] Alelign A., Tekeste Z., Petros B. (2018). Prevalence of malaria in Woreta town, Amhara region, Northwest Ethiopia over eight years. *BMC Public Health*.

[33] Negatu G., Abebe G., Yalew W. (2020). Prevalence of malaria and its associated factors among malaria suspected patients attending at Hamusite Health Center, Northwest Ethiopia a Cross-Sectional Study. *Journal of Parasitology Research*.

[34] Tadesse F., Fogarty A., Deressa W. (2018). Prevalence and associated risk factors of malaria among adults in East Shewa Zone of Oromia Regional State, Ethiopia: a cross-sectional study. *BMC Public Health*.

[35] Haji Y., Fogarty A. W., Deressa W. (2016). Prevalence and associated factors of malaria among febrile children in Ethiopia: a cross-sectional health facility-based study. *Acta Tropica*.

[36] Abossie A., Yohanes T., Nedu A., Tafesse W., Damitie M. (2020). Prevalence of malaria and associated risk factors among febrile children under five years: a cross-sectional study in Arba Minch Zuria district, South Ethiopia. *Infection and Drug Resistance*.

[37] Gontie G., Wolde H., Baraki A. (2020). Prevalence and associated factors of malaria among pregnant women in Sherkole district, Benishangul Gumuz regional state, West Ethiopia. *BMC Infectious Diseases*.

[38] Debo G., Kassa D. (2016). Prevalence of malaria and associated factors in Benna Tsemay district of pastoralist community, Southern Ethiopia. *Tropical Diseases Travel Medicine and Vaccines*.

[39] Tarekegn M., Tekie H., Dugassa S., Wolde-H Y. (2021). Malaria prevalence and associated risk factors in Dembiya district, North-western Ethiopia. *Malaria Journal*.

[40] Belay B., Tegenu G., Araya G. (2020). *Malaria Prevalence and Knowledge, Attitude and Practice about Malaria among Febrile Patients Attending Chagni Health Center. Northwest Ethiopia: A Cross-Sectional Study*.

